# HIV infection is associated with elevated biomarkers of immune activation in Ugandan adults with pneumonia

**DOI:** 10.1371/journal.pone.0216680

**Published:** 2019-05-15

**Authors:** Richard J. Wang, Julia Moore, Daniela Moisi, Emily G. Chang, Patrick Byanyima, Sylvia Kaswabuli, Emmanuel Musisi, Ingvar Sanyu, Abdulwahab Sessolo, Rejani Lalitha, William Worodria, J. Lucian Davis, Kristina Crothers, Jue Lin, Michael M. Lederman, Peter W. Hunt, Laurence Huang

**Affiliations:** 1 Department of Medicine, University of California San Francisco, San Francisco, California, United States of America; 2 Department of Medicine, Case Western Reserve University School of Medicine, Cleveland, Ohio, United States of America; 3 Department of Statistics, University of California Davis, Davis, California, United States of America; 4 Makerere University – University of California San Francisco Research Collaboration, Infectious Diseases Research Collaboration, Kampala, Uganda; 5 Department of Internal Medicine, Makerere College of Health Sciences, Kampala, Uganda; 6 Department of Internal Medicine, Yale School of Medicine, New Haven, Connecticut, United States of America; 7 Department of Medicine, University of Washington School of Medicine, Seattle, Washington, United States of America; 8 Department of Biochemistry and Biophysics, University of California San Francisco, San Francisco, California, United States of America; University of Pittsburgh Centre for Vaccine Research, UNITED STATES

## Abstract

**Introduction:**

Pneumonia is an important cause of morbidity and mortality in persons living with human immunodeficiency virus (HIV) infection. How immune activation differs among HIV-infected and HIV-uninfected adults with pneumonia is unknown.

**Methods:**

The Inflammation, Aging, Microbes, and Obstructive Lung Disease (I AM OLD) Cohort is a prospective cohort of adults with pneumonia in Uganda. In this cross-sectional analysis, plasma was collected at pneumonia presentation to measure the following 12 biomarkers: interleukin 6 (IL-6), soluble tumor necrosis factor receptors 1 and 2 (sTNFR-1 and sTNFR-2), high sensitivity C-reactive protein (hsCRP), fibrinogen, D-dimer, soluble CD27 (sCD27), interferon gamma-inducible protein 10 (IP-10), soluble CD14 (sCD14), soluble CD163 (sCD163), hyaluronan, and intestinal fatty acid binding protein. We asked whether biomarker levels differed between HIV-infected and HIV-uninfected participants, and whether higher levels of these biomarkers were associated with mortality.

**Results:**

One hundred seventy-three participants were enrolled. Fifty-three percent were HIV-infected. Eight plasma biomarkers—sTNFR-1, sTNFR-2, hsCRP, D-dimer, sCD27, IP-10, sCD14, and hyaluronan—were higher among participants with HIV infection, after adjustment for pneumonia severity. Higher levels of 8 biomarkers—IL-6, sTNFR-1, sTNFR-2, hsCRP, IP-10, sCD14, sCD163, and hyaluronan—were associated with increased 2-month mortality.

**Conclusions:**

As in other clinical contexts, HIV infection is associated with a greater degree of immune activation among Ugandan adults with pneumonia. Some of these are also associated with short-term mortality. Further study is needed to explore whether these biomarkers might predict poor long-term outcomes—such as the development of obstructive lung disease—in patients with HIV who have recovered from pneumonia.

## Introduction

Although infections by opportunistic pathogens have declined with uptake of antiretroviral therapy (ART), pneumonia remains an important cause of morbidity and mortality in persons living with human immunodeficiency virus (HIV) infection. HIV-infected persons have a higher risk of pneumonia compared to HIV-uninfected persons.[1–3] Among persons with HIV infection, the occurrence of pneumonia is associated with increased risk of airway obstruction,[4] lung cancer,[5] and death.[6]

Extensive prior research has documented that HIV infection is associated with abnormal immune activation.[7] Biomarkers of inflammation,[8] interferon response,[9] T-cell activation,[10] and monocyte and macrophage activation[11,12] have been shown to be increased among HIV-infected persons compared to uninfected persons, some of which persist even after virologic suppression with ART. Furthermore, persistent immune activation in the setting of ART-mediated viral suppression has been associated with several chronic morbidities—including cardiovascular disease,[13] neurocognitive dysfunction,[14] and diabetes[15]—and mortality.[16–18]

HIV infection is also independently associated with chronic obstructive pulmonary disease (COPD)[19] and, among HIV-infected persons, a history of pneumonia is an established risk factor for airway obstruction.[4] It is unknown whether abnormal immune activation may play a role in mediating a potential causal relationship between HIV infection, pneumonia, and the development of COPD. To our knowledge, no prior study has investigated differences in immune activation between HIV-infected and HIV-uninfected persons in the setting of pneumonia.

To better understand the association between HIV infection and immune activation in the setting of pneumonia, we conducted a cross-sectional study to compare markers of immune activation between HIV-infected and HIV-uninfected Ugandan adults with pneumonia. Twelve markers were selected to evaluate different aspects of immune activation: inflammation (interleukin 6, soluble tumor necrosis factor receptor 1, soluble tumor necrosis factor receptor 2, and high sensitivity C-reactive protein), coagulation (fibrinogen and D-dimer), T-cell activation (soluble CD27), interferon response (interferon gamma-inducible protein 10), macrophage and monocyte activation (soluble CD14 and soluble CD163), fibrosis (hyaluronan), and gut epithelial integrity (intestinal fatty acid binding protein). These biomarkers were selected because they have previously been shown to be elevated or to be predictive of poor outcomes, or both, among HIV-infected persons.[13–17,20–22] Our goals were to determine whether biomarker levels differed between HIV-infected and HIV-uninfected participants with pneumonia, and whether higher levels of these biomarkers were associated with increased 2-month mortality.

## Methods

### Study design

This is a cross-sectional analysis of baseline characteristics of a cohort of adults with pneumonia in Uganda.

### Study population

The Inflammation, Aging, Microbes, and Obstructive Lung Disease (I AM OLD) Cohort is a prospective cohort study of adults with pneumonia in Uganda. Adult patients 18 years or older who presented with suspected pneumonia to the China-Uganda Friendship Hospital Naguru, an urban district-level hospital in Kampala, Uganda, were screened for enrollment. Patients were eligible for inclusion if they complained of cough and had an abnormal chest radiograph determined by one of two pulmonologists (RL or WW) to be consistent with a pneumonia. Patients were excluded if they were pregnant or unable to provide consent.

### Measurements

Demographic, clinical, and laboratory data were collected using standardized case report forms at the time of enrollment. Data that were collected included reported symptoms, vital signs, and oxygen saturation by pulse oximetry. Functional status was ascertained by asking participants what percentage of their day was spent in bed. Participants were categorized as bedbound if they reported spending greater than 50 percent of their day in bed. If a participant reported known HIV infection, data regarding ART and trimethoprim/sulfamethoxazole *Pneumocystis* pneumonia prophylaxis were recorded, and a blood sample was collected for CD4 count. If a participant reported unknown or negative HIV status, the participant was offered HIV testing and, if positive, a blood sample was collected for CD4 count. Sputum was obtained from all patients for testing for tuberculosis (TB) by smear microscopy, molecular testing by GeneXpert MTB/RIF (Cepheid, Sunnyvale, California, United States), and mycobacterial culture with Lowenstein-Jensen and Mycobacterial Growth Indicator Tubes. HIV-infected participants whose sputa were negative for acid-fast bacilli on smear microscopy and negative for tuberculosis by GeneXpert MTB/RIF underwent bronchoscopy if clinically indicated. During bronchoscopy, the tracheobronchial tree was inspected for lesions consistent with Kaposi’s sarcoma, and bronchoalveolar lavage (BAL) samples were obtained. BAL samples were inspected by microscopy for *Pneumocystis jirovecii* using a modified Giemsa stain. BAL samples were also tested for tuberculosis by smear microscopy and by mycobacterial culture with Lowenstein-Jensen and Mycobacterial Growth Indicator Tubes.

Blood samples for all participants were collected for biomarker measurement via peripheral venipuncture, from which plasma was extracted and cryopreserved at -80°C before being shipped on dry ice to the United States. Twelve plasma biomarkers were selected for measurement by immunoassay to assess for immune activation. As mentioned previously, the selected biomarkers were: interleukin 6 (IL-6), soluble tumor necrosis factor receptor 1 (sTNFR-1), soluble tumor necrosis factor receptor 2 (sTNFR-2), high sensitivity C-reactive protein (hsCRP), fibrinogen, D-dimer, soluble CD27 (sCD27), interferon gamma-inducible protein 10 (IP-10, also known as CXCL10), soluble CD14 (sCD14), soluble CD163 (sCD163), hyaluronan, and intestinal fatty acid binding protein (IFABP). The biomarkers were measured using ELISA kits obtained from R&D Systems (for IL-6, sTNFR-1, sTNFR-2, hsCRP, IP-10, sCD14, sCD163, hyaluronan, and IFABP), Diagnostica Stago (for D-dimer), and Abcam (for fibrinogen). Study personnel who performed biomarker measurements were blinded to clinical information, including HIV status.

Patients or their surrogates were contacted by phone two months after enrollment to ascertain vital status.

### Statistical analysis

All data were analyzed with STATA 15.1 (StataCorp, College Station, Texas, United States).

Baseline characteristics were compared between HIV-infected and HIV-uninfected participants using Fisher’s exact test for categorical variables and the Mann-Whitney *U* test for continuous variables.

The primary analytic goal was to compare biomarker levels between HIV-infected and HIV-uninfected participants. Because the distributions of biomarkers did not meet assumptions of normality, the nonparametric Mann-Whitney *U* test was used for comparisons.

We then evaluated whether differences in biomarker levels were independent of severity of pneumonia. To do so, 5 variables known to be associated with pneumonia severity were selected for adjustment; these were age, heart rate, respiratory rate, oxygen saturation, and whether or not a participant was, by self-report, bedbound.[23,24] Initially, to determine whether differences in biomarker levels were independent of pneumonia severity, we fit a multiple linear regression model for each biomarker as a continuous outcome in which HIV status (infected or uninfected) was the predictor of interest, and co-variates were age, heart rate, respiratory rate, oxygen saturation, and bedbound status. However, regression diagnostics suggested that for several biomarkers, model residuals were not normally distributed and that variance differed substantially between the HIV-infected and HIV-uninfected subgroups. To satisfy modelling assumptions, biomarker levels were log_10_-transformed, and log-linear models were then fitted using the same predictors. The adjusted percentage difference between HIV-infected and HIV-uninfected participants for each biomarker was calculated by exponentiating the model coefficients for HIV status and then plotted graphically. The log_10_-transformed biomarker levels were then further transformed by dividing by the interquartile range (IQR), and linear models were again constructed with the same predictor variables. Dividing by the IQR effectively standardized each biomarker to its own range of test values, allowing for comparison of the relative strength of association between HIV infection and each biomarker. To test whether pneumonia etiology might contribute to differences in biomarker levels, we conducted a sensitivity analysis by generating models restricted only to participants with TB infection.

We then evaluated whether biomarker levels were predictive of 2-month mortality. For each biomarker measured, the cohort was divided into tertiles. A risk ratio was calculated to compare the risk of mortality for participants with values in the highest tertile to the risk of mortality for participants in the lower two tertiles.

### Ethical issues

Ethical approval was obtained from the Makerere University School of Medicine Research and Ethics Committee, the Uganda National Council for Science and Technology, and the University of California San Francisco Committee on Human Research. All study participants were informed about the study by a nurse or a physician and provided written informed consent before participation. Disease management was conducted by staff physicians at the China-Uganda Friendship Hospital Naguru.

## Results

### Characteristics of the study population

One hundred seventy-three participants were enrolled in the I AM OLD cohort between October 6, 2015 and July 20, 2017. Table 1 summarizes their baseline characteristics. Forty-five percent of participants were women and the median age for all participants was 33 years. Overall, 91 (52.6%) participants were HIV-infected. Sixty-seven participants had a prior diagnosis of HIV infection, 29 (43.2%) of whom were on combination anti-retroviral therapy at the time of presentation. All other participants agreed to be tested for HIV infection; 24 additional participants were newly diagnosed with HIV infection and 82 were determined to be HIV negative. Among the 91 participants with HIV infection, the median CD4 count was 134 cells/μL and nearly two-thirds had a CD4 count less than 200 cells/μL.

**Table 1:** Baseline characteristics, by HIV status.

Variable, *n* (%) or median [IQR]	HIV uninfected *n* = 82	HIV infected *n* = 91	*p*-value
**HIV-related variables**			
New diagnosis of HIV		24 (26.4)	
On ART		29 (31.9)	
On PCP^†^ prophylaxis		42 (46.2)	
CD4 count (*n* = 90)		134 [56–327]	
Newly diagnosed with HIV (*n* = 24)		139 [54–293]	
Known HIV, on ART (*n* = 28)		127 [58–350]	
Known HIV, not on ART (*n* = 38)		138 [57–252]	
CD4 count < 200 (*n* = 90)		57 (63.3)	
**Demographics**			
Female	33 (40.2)	44 (48.4)	0.36
Age	31.7 [26.6–40.4]	33.8 [29.1–44.3]	0.02
**Symptoms**			
Cough	82 (100.0)	91 (100.0)	-
Fever	67 (81.7)	86 (94.5)	0.02
Weight loss	65 (79.3)	87 (95.6)	0.002
Sputum production	69 (84.2)	77 (84.6)	1
Shortness of breath	47 (57.3)	55 (60.4)	0.76
Chest pain	62 (75.6)	56 (61.5)	0.05
Spending > 50% of day in bed	10 (12.2)	26 (28.6)	0.009
**Exposures**			
Current tobacco use	8 (9.8)	6 (6.7)	0.58
Current alcohol use	36 (43.9)	38 (41.8)	0.88
Received antibiotics previously	70 (85.4)	70 (76.9)	0.18
**Baseline vital signs**			
Heart rate	100 [81–115]	108 [91–124]	0.008
Heart rate > 120	13 (15.9)	28 (30.8)	0.03
Respiratory rate	22 [20–24]	24 [20–28]	0.03
Respiratory rate > 30	6 (7.3)	18 (19.8)	0.04
Oxygen saturation	96 [94–97]	95 [92–97]	0.10
Oxygen saturation < 90%	6 (7.3)	16 (17.6)	0.07
**Diagnosed with tuberculosis**	48 (58.5)	52 (57.1)	0.88
**Diagnosed with PCP**^†^		2 (2.2)	

Fisher’s Exact Test was used for categorical variables, and Mann-Whitney *U* Test was used for continuous variables.

^†^ *Pneumocystis* pneumonia

Participants with HIV infection were older, more likely to report fever and weight loss, and more likely to be bedbound. They also had higher heart rates and respiratory rates. Participants with HIV infection were more likely to have an oxygen saturation below 90% while breathing room air (17.6% compared to 7.3%, *p* = 0.07). One hundred (57.8%) of the 173 participants were diagnosed with TB. There was no significant difference in the proportion of patients diagnosed with TB among HIV-infected patients than among HIV-uninfected patients (57.1% compared to 58.5%, *p* = 0.88). Among HIV-infected participants, only two were found to have *Pneumocystis* pneumonia.

### Comparison of biomarker levels between HIV-infected and HIV-uninfected participants

Table 2 reports the median biomarker levels for participants with HIV infection and participants without HIV infection. Plasma levels of all biomarkers other than fibrinogen and IFABP were significantly higher for HIV-infected participants than for HIV-uninfected participants; the differences persisted for all biomarkers regardless of whether or not the HIV-infected participants were on ART at the time of enrollment, except for sCD163. Levels of sCD163 were significantly higher for HIV-infected participants on ART compared to HIV-uninfected participants, but not for HIV-infected participants not on ART (S1 Table).

**Table 2:** Median biomarker measurements, by HIV status.

Biomarkers, median [IQR]	HIV-uninfected	HIV-infected	*p*-value
IL-6 (pg/mL)	14.0 [3.1–34.4]	25.6 [8.7–56.5]	0.002
sTNFR-1 (ng/mL)	1.49 [1.04–2.06]	2.41 [1.62–3.64]	<0.0001
sTNFR-2 (ng/mL)	3.81 [3.01–6.17]	6.67 [5.10–13.19]	<0.0001
hsCRP (μg/mL)	20.1 [5.9–61.2]	59.5 [19.4–141.8]	0.0007
Fibrinogen (mg/mL)	6.25 [4.25–9.76]	7.77 [4.33–11.9]	0.22
D-dimer (ng/mL)	574 [317–1,504]	1,079 [576–2,300]	0.0001
sCD27 (U/mL)	37.6 [17.7–56.3]	55.3 [29.3–106.5]	0.0003
IP-10 (ng/mL)	0.51 [0.20–1.01]	1.14 [0.57–1.73]	<0.0001
sCD14 (μg/mL)	2.24 [1.76–2.87]	3.64 [2.77–4.55]	<0.0001
sCD163 (ng/mL)	776 [508–1,399]	1,014 [623–1,544]	0.03
Hyaluronan (ng/mL)	37.4 [22.7–60.2]	72.6 [35.9–147.4]	<0.0001
IFABP (ng/mL)	0.94 [0.59–1.58]	0.82 [0.36–1.29]	0.18

Differences were tested by Mann-Whitney *U* Test.

**Fig 1:**
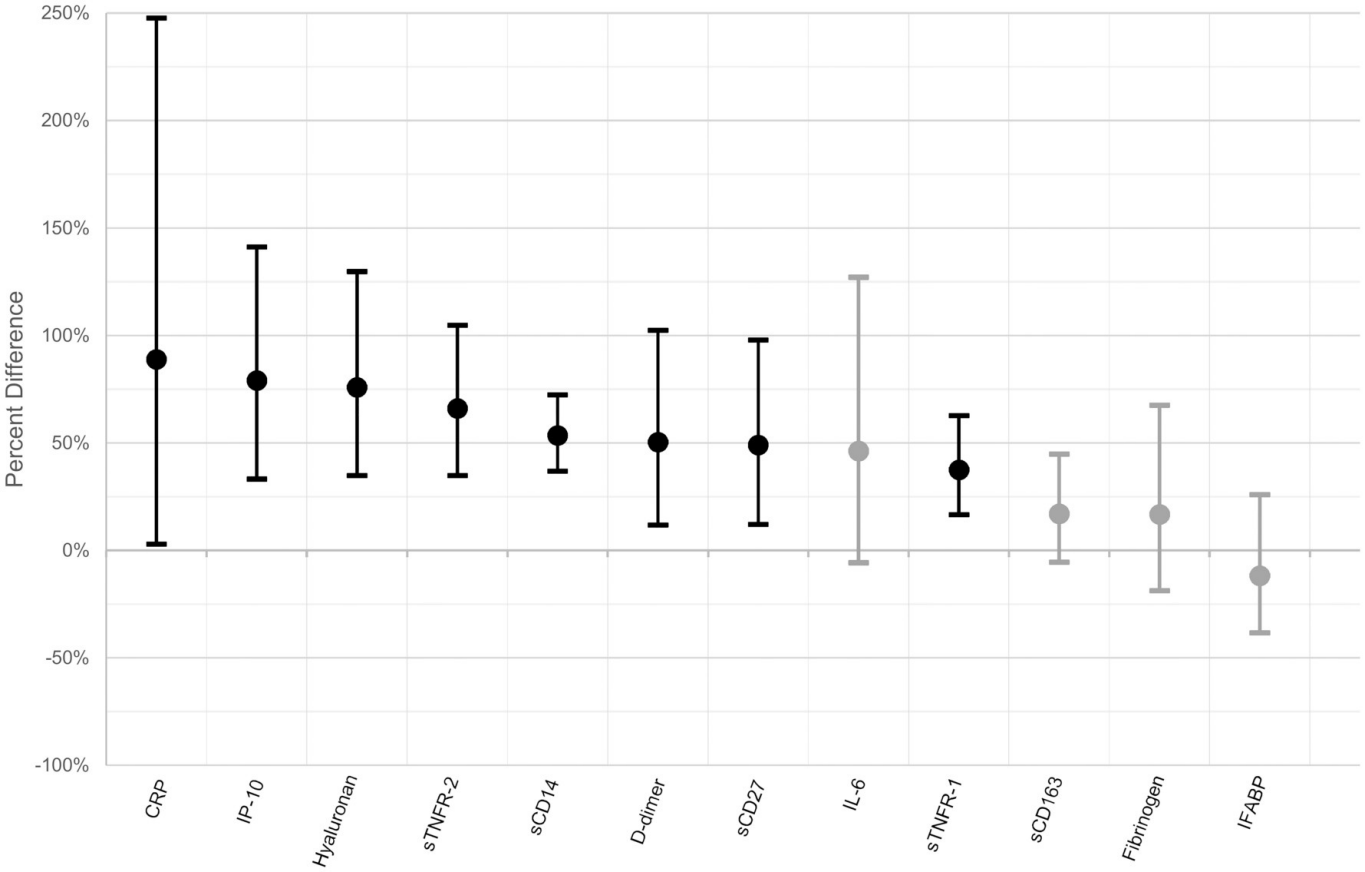
Adjusted percentage difference in mean biomarker levels comparing HIV-infected participants with HIV-uninfected participants. Each point represents the adjusted percentage difference in mean biomarker levels comparing participants with HIV infection to participants without HIV infection, as estimated by log-linear regression models controlling for age, heart rate, respiratory rate, oxygen saturation, and bedbound status. The bars represent the 95% confidence intervals for the estimate. In cases when the bounds of the 95% confidence interval include 0, the points and bars have been shaded in gray. The graphic can be interpreted as per this example: after adjustment for age, heart rate, respiratory rate, oxygen saturation, and bedbound status, the plasma concentration of IP-10 was 79% higher in participants with HIV infection than in participants without HIV infection, with a 95% confidence interval of 33% to 141%.

To test whether the relationships between HIV infection and plasma biomarkers were independent of pneumonia severity, multiple linear regression models were fitted to adjust for variables that are known to be associated with pneumonia severity: age, heart rate, respiratory rate, oxygen saturation, and bedbound status. After adjustment, HIV infection remained significantly associated with higher plasma concentrations of sTNFR-1, sTNFR-2, hsCRP, D-dimer, sCD27, IP-10, sCD14, and hyaluronan. Fig 1 illustrates the adjusted percentage difference in mean biomarker levels comparing participants with HIV infection to participants without HIV infection. For example, after adjustment for pneumonia severity, the plasma concentration of IP-10 was 79% higher in participants with HIV infection than in participants without HIV infection. Although HIV infection was significantly associated with higher plasma concentrations of IL-6 and sCD163 in the univariate analysis, these associations were attenuated with adjustment and were no longer statistically significant. Differences in the plasma concentration of fibrinogen and IFABP between HIV-infected and HIV-uninfected participants did not meet statistical significance in either the univariate or multivariate analyses.

To allow for comparison of the strength of association between HIV infection and each plasma biomarker, each log_10_-transformed biomarker was standardized by dividing by its own IQR. Multiple linear regression models were again fitted to adjust for pneumonia severity, and the coefficients of association between HIV infection and each biomarker are reported in Table 3. After standardization for dynamic range of testing, HIV infection was most strongly associated with sCD14, followed by sTNFR-2, hyaluronan, and IP-10.

**Table 3:** Coefficient estimates for the effect of HIV status on log_10_-transformed plasma biomarker level divided by interquartile range, after adjustment for age, heart rate, respiratory rate, oxygen status, and functional status with multiple linear regression modeling.

Biomarker	Coefficient	95% confidence interval	*p*-value
Interleukin 6	0.185	(-0.029, 0.398)	0.09
Soluble TNF Receptor 1	0.371	(0.178, 0.565)	<0.001
Soluble TNF Receptor 2	0.594	(0.348, 0.840)	<0.001
High sensitivity C-reactive protein	0.289	(0.122, 0.565)	0.04
Fibrinogen	0.155	(-0.211, 0.522)	0.40
D-dimer	0.303	(0.082, 0.524)	0.007
Soluble CD27	0.326	(0.093, 0.559)	0.007
IFN-γ-Inducible Protein 10	0.427	(0.209, 0.645)	<0.001
Soluble CD14	0.712	(0.520, 0.904)	<0.001
Soluble CD163	0.163	(-0.060, 0.386)	0.15
Hyaluronan	0.447	(0.235, 0.659)	<0.001
Intestinal Fatty Acid Binding Protein	-0.109	(-0.414, 0.197)	0.48

To assess whether the association between HIV infection and elevated biomarkers would be affected by pneumonia etiology, we conducted a sensitivity analysis in which we restricted the IQR-standardized models only to participants diagnosed with tuberculosis which was the predominant pneumonia etiology in this cohort. When restricting analysis only to participants diagnosed with tuberculosis, the associations of hsCRP and D-dimer with HIV infection were attenuated and no longer significant; the other associations between HIV infection and biomarker levels were preserved (S2 Table).

### Biomarkers and 2-month mortality

Of the 173 participants included in the analysis, 151 (87.2%) were alive at 2 months, 13 (7.5%) had died, and 9 (5.2%) were lost to follow-up. Of the 13 participants who died, 9 were HIV-infected and 4 were HIV-uninfected. Mortality for HIV-infected participants was higher than for HIV-uninfected participants (9.9% compared to 4.9%, *p* = 0.25) but this difference was not statistically significant. Of the 9 participants lost to follow-up, 8 were HIV-infected and 1 was HIV-uninfected. HIV-infected participants were more likely to be lost to follow-up (8.8% compared to 1.2%, *p* = 0.04). Biomarker levels were not significantly different when comparing participants lost to follow-up with participants known to be alive at 2 months after enrollment (S3 Table).

Biomarker association with mortality was compared for the 164 participants with known 2-month vital status. When comparing participants with biomarker levels in the highest tertile to participants with biomarker levels in the lower two tertiles, higher levels of IL-6, sTNFR-1, sTNFR-2, hsCRP, IP-10, sCD14, sCD163, and hyaluronan were significantly associated with increased risk of mortality (Table 4). In a stratified analysis, we did not detect any effect modification by HIV status, although this study was not sufficiently powered to do so (S4 Table).

**Table 4:** Risk ratios comparing the risk of mortality for the highest tertile compared to lower tertiles for each biomarker.

Biomarker	Risk Ratio	95% confidence interval	*p*-value
High sensitivity C-reactive protein	6.13	(1.74, 21.6)	0.002
Soluble CD14	4.71	(1.52, 14.6)	0.005
Soluble TNF Receptor 1	4.71	(1.52, 14.6)	0.005
IFN-γ-Inducible Protein 10	4.34	(1.40, 13.5)	0.01
Interleukin 6	4.04	(1.27, 12.8)	0.02
Soluble TNF Receptor 2	3.44	(1.18, 10.0)	0.03
Hyaluronan	3.35	(1.15, 9.75)	0.03
Soluble CD163	3.29	(1.13, 9.57)	0.03
D-dimer	1.23	(0.42, 3.57)	0.76
Intestinal Fatty Acid Binding Protein	1.01	(0.32, 3.18)	1
Fibrinogen	0.98	(0.31, 3.08)	1
Soluble CD27	0.86	(0.23, 3.16)	1

Differences in risk of death were tested by Fisher’s Exact Test.

## Discussion

We measured the levels of 12 biomarkers of immune activation in plasma samples obtained from Ugandan adults with pneumonia, of whom 53% were HIV-infected and 58% were diagnosed with TB. We found that plasma concentrations of 10 of the 12 biomarkers were significantly higher among participants with HIV infection, and that 8 of the 12 biomarkers remained significantly higher even after adjustment for pneumonia severity. These 8 biomarkers were sTNFR-1, sTNFR-2, hsCRP, D-dimer, sCD27, IP-10, sCD14, and hyaluronan; of these, sCD14, sTNFR-2, hyaluronan, and IP-10 were most strongly associated with HIV infection.

The 8 biomarkers associated with HIV infection in this cohort reflect the activity of multiple biologic pathways, including inflammation (sTNFR-1, sTNFR-2, hsCRP), coagulation (D-dimer), T-cell activation (sCD27), interferon response (IP-10), monocyte and macrophage activation (sCD14), and fibrosis (hyaluronan). Collectively, these data indicate that HIV infection is associated with a greater degree of immune activation even in the context of an acute infection like pneumonia. This is consistent with previous research that has found that, in other clinical contexts, plasma levels of these biomarkers are elevated in persons with HIV infection compared to uninfected controls and that, even in the setting of treatment with ART, abnormal immune activation persists in HIV-infected individuals.[8,9,25–29]

We also conducted an analysis of whether these plasma biomarkers were predictive of mortality in a population of adults with pneumonia that is unique because of a high prevalence of HIV and TB infection. We found that 8 of the 12 biomarkers were predictive of short-term mortality after pneumonia. These were IL-6, sTNFR-1, sTNFR-2, hsCRP, IP-10, sCD14, sCD163, and hyaluronan. To our knowledge, this is the first time that mortality after pneumonia has been shown to be associated with sTNFR-1, sTNFR-2, sCD14, IP-10, and hyaluronan; these may be candidate biomarkers for future studies to ascertain their predictive utility.

Our study has several important limitations. The cross-sectional study design limits our ability to draw causal inferences. Specifically, the cross-sectional study design limits our ability to implicate immune activation as a mediator of poor long-term outcomes, such as obstructive lung disease, among HIV-infected patients who recover from pneumonia; longitudinal prospective cohort studies may be instrumental to answering this question. Also, since plasma was obtained at the time of clinical presentation, we cannot ascertain whether these biomarkers were elevated prior to the onset of pneumonia—and perhaps may have had a role in the pathogenesis of pneumonia—or whether these biomarkers reflect a differential host response to pneumonia in the setting of HIV infection. There is evidence to support both explanations. For example, in both HIV-infected and HIV-uninfected persons, higher levels of hsCRP were predictive of increased risk for developing pneumonia.[30,31] On the other hand, several biomarkers that were tested, such as IL-6 and hsCRP, are known to be part of the normal inflammatory response to an infection, such as pneumonia, and may be dysregulated in the setting of HIV infection.[32,33] It seems likely that both explanations contribute to some degree to the differences in biomarker levels observed in this study, but teasing apart the causal relationship is beyond the capacity of a cross-sectional study.

A second limitation regards our ability to adjust completely for pneumonia severity. Analysis of baseline characteristics indicates that, in general, participants with HIV infection likely had more severe pneumonia, as evidenced by statistically significant differences in vital signs (heart rate and respiratory rate) and bedbound status. It is possible that differences in biomarker levels between participants with and without HIV infection might be at least partially attributable to differences in disease severity that we were unable to measure and adjust for in our analysis. Higher plasma levels of hsCRP and D-dimer have been shown to be associated with mortality in patients with pneumonia.[34–40] Plasma hyaluronan has been shown to be associated with mortality in patients with sepsis.[41–43] In our analysis, we attempted to adjust for the severity of pneumonia, but our ability to do so is likely incomplete. However, we also note the possibility that abnormal immune activation and an inflammatory milieu may mediate the effect of HIV infection on pneumonia severity, in which case adjustment for disease severity may not be appropriate and may in fact attenuate the true association between HIV infection and plasma biomarker levels.

Thirdly, there was limited data obtained in this study regarding pneumonia etiology, other than tuberculosis, and it is possible that differences in pneumonia etiology between HIV-infected and HIV-uninfected participants may account for some degree of difference in biomarker levels. Specifically, opportunistic infections, such as *Pneumocystis jirovecii* are known to elicit a strong host inflammatory response.[44,45] However, only two HIV-infected participants in this study were diagnosed with *Pneumocystis* pneumonia. Furthermore, a recent cohort study in Malawi of a similar population of adult patients with pneumonia revealed that, after tuberculosis, the most common etiology of pneumonia was *Streptococcus pneumoniae*, followed by respiratory viruses, in both HIV-infected and HIV-uninfected patients.[46] Therefore, the likelihood of substantial differences in pneumonia etiology between HIV-infected and HIV-uninfected participants is small and we do not think that this possibility invalidates our results.

A fourth limitation regards the validity of extending the conclusions of this study to other populations. The high proportion (> 50%) of participants with TB is unique in this cohort, and the findings of this study may not reflect the biology of other populations of adults with pneumonia, in which the proportion of participants with TB may not be as high. Also, HIV-infected participants in this cohort are notable for the relatively low uptake of ART and low CD4 counts. While this is not unusual in sub-Saharan Africa—the median CD4 count in a similar cohort of Malawian patients with pneumonia was 99, compared to 134 in this study[46]—it is likely that the relationship between several of these biomarkers and HIV infection may be attenuated in other HIV populations with a higher proportion of viral suppression; indeed, HIV viral load has been shown to be associated with increased levels of IP-10 and sTNFR-2.[47,48]

Despite these limitations, we believe that this study represents a substantial contribution to the literature. While biomarkers of immune activation have been studied extensively amongst HIV-infected persons, few studies have measured them amongst HIV-infected and HIV-uninfected persons with a concurrent acute infection, such as pneumonia. Our novel finding that participants with HIV infection have a greater degree of immune activation, even in the setting of a pneumonia, confirms and extends prior research on the persistence of immune activation among HIV-infected persons, including those who are virally suppressed on ART.

In summary, we measured 12 plasma biomarkers of immune activation in a cohort of Ugandan adults diagnosed with pneumonia, 53% of whom were infected with HIV and 58% of whom had TB. Plasma levels for 8 of these biomarkers—sTNFR-1, sTNFR-2, hsCRP, D-dimer, IP-10, sCD14, sCD27, and hyaluronan—were higher among participants with HIV infection as compared to those without HIV infection, after adjustment for pneumonia severity. These results suggest that, among adults with pneumonia, concurrent HIV infection is associated with a greater degree of immune activation. Furthermore, higher levels of 8 biomarkers—IL-6, sTNFR-1, sTNFR-2, hsCRP, IP-10, sCD14, sCD163, and hyaluronan—were associated with increased 2-month mortality. Further study is needed to explore whether these biomarkers might predict poor long-term outcomes—such as the development of obstructive lung disease—in patients with HIV who have recovered from pneumonia.

## Supporting information

S1 Table Median biomarker measurements, by HIV status and ART use. (PDF)

S2 Table Sensitivity analysis comparing coefficient estimates for the effect of HIV status on log_10_-transformed plasma biomarker level divided by interquartile range, after adjustment for age, heart rate, respiratory rate, oxygen status, and functional status with multiple linear regression modeling for the entire cohort to estimates for only those participants diagnosed with tuberculosis. (PDF)

S3 Table Median biomarker measurements, comparing participants known to be alive at 2 months and participants lost to follow-up. Differences were tested by Mann-Whitney *U* Test.
(PDF)

S4 Table Risk ratios, with 95% confidence intervals, comparing the risk of mortality for the highest tertile compared to lower tertiles for each biomarker, stratified by HIV. (PDF)
