# Supplementary material for: HIV infection is associated with elevated biomarkers of immune activation in Ugandan adults with pneumonia
Source: PLoS One. 2019 May 15;14(5):e0216680. doi: 10.1371/journal.pone.0216680 (PMC6519791; doi:10.1371/journal.pone.0216680)
Supplement: S1 Table — (PDF) [file pone.0216680.s001.pdf]

S1 Table. Median biomarker measurements, by HIV status and ART use

Biomarkers, median [IQR]	HIV-uninfected (*n* = 82)		HIV-infected, on ART (*n* = 29)		*p*-value*	HIV-infected, not on ART (*n* = 62)		*p*-value*
IL-6 (pg/mL)	14.0	[3.1 – 34.4]	43.2	[10.1 – 87.3]	0.002	23.2	[8.1 – 44.5]	0.03
sTNFR-1 (ng/mL)	1.49	[1.04 – 2.06]	2.25	[1.51 – 4.22]	0.001	2.43	[1.65 – 3.44]	<0.001
sTNFR-2 (ng/mL)	3.81	[3.01 – 6.17]	6.36	[4.76 – 17.5]	<0.001	7.31	[5.38 – 11.8]	<0.001
hsCRP (µg/mL)	20.1	[5.9 – 61.2]	88.4	[23.5 – 172.7]	0.002	45.4	[15.1 – 135.9]	0.01
Fibrinogen (mg/mL)	6.25	[4.25 – 9.76]	8.41	[3.23 – 14.2]	0.40	7.58	[4.42 – 11.9]	0.27
D-dimer (ng/mL)	574	[317 – 1,504]	1,082	[806 – 2,194]	0.001	1,061	[479 – 2,650]	0.002
sCD27 (U/mL)	37.6	[17.7 – 56.3]	46.9	[27.6 – 94.4]	0.05	63.8	[29.3 – 122.6]	<0.001
IP-10 (ng/mL)	0.51	[0.20 – 1.01]	1.59	[0.55 – 2.34]	<0.001	1.01	[0.59 – 1.71]	<0.001
sCD14 (µg/mL)	2.24	[1.76 – 2.87]	4.23	[3.66 – 5.73]	<0.001	3.23	[2.64 – 4.33]	<0.001
sCD163 (ng/mL)	776	[508 – 1,399]	1,094	[695 – 1,589]	0.02	928	[595 – 1,526]	0.12
Hyaluronan (ng/mL)	37.4	[22.7 – 60.2]	55.1	[32.8 – 116.4]	0.01	80.9	[36.1 – 151.5]	<0.001
IFABP (ng/mL)	0.94	[0.59 – 1.58]	0.98	[0.65 – 27.6]	0.62	0.59	[0.29 – 1.48]	0.14

* compared with HIV-uninfected participants, differences tested by Mann-Whitney *U* Test
