# Supplementary material for: HIV infection is associated with elevated biomarkers of immune activation in Ugandan adults with pneumonia
Source: PLoS One. 2019 May 15;14(5):e0216680. doi: 10.1371/journal.pone.0216680 (PMC6519791; doi:10.1371/journal.pone.0216680)
Supplement: S2 Table — (PDF) [file pone.0216680.s002.pdf]

S2 Table. Sensitivity analysis comparing coefficient estimates for the effect of HIV status on log_{10}-transformed plasma biomarker level divided by interquartile range, after adjustment for age, heart rate, respiratory rate, oxygen status, and functional status with multiple linear regression modeling for the entire cohort to estimates for only those participants diagnosed with tuberculosis

Biomarker	Entire cohort		Only TB	
	Coefficient	*p*-value	Coefficient	*p*-value
Interleukin 6	0.185	0.09	0.212	0.09
Soluble TNF Receptor 1	0.371	<0.001	0.388	0.001
Soluble TNF Receptor 2	0.594	<0.001	0.652	<0.001
High sensitivity C-reactive protein	0.289	0.04	0.062	0.70
Fibrinogen	0.155	0.40	0.076	0.30
D-dimer	0.303	0.007	0.190	0.17
Soluble CD27	0.326	0.007	0.367	0.02
IFN-γ-Inducible Protein 10	0.427	<0.001	0.387	0.005
Soluble CD14	0.712	<0.001	0.725	<0.001
Soluble CD163	0.163	0.15	0.223	0.13
Hyaluronan	0.447	<0.001	0.591	<0.001
Intestinal Fatty Acid Binding Protein	-0.109	0.48	-0.156	0.46
