# Supplementary material for: HIV infection is associated with elevated biomarkers of immune activation in Ugandan adults with pneumonia
Source: PLoS One. 2019 May 15;14(5):e0216680. doi: 10.1371/journal.pone.0216680 (PMC6519791; doi:10.1371/journal.pone.0216680)
Supplement: S3 Table — Differences were tested by Mann-Whitney U Test. (PDF) [file pone.0216680.s003.pdf]

S3 Table. Median biomarker measurements, comparing participants known to be alive at 2 months and participants lost to follow-up

**Biomarkers, median [IQR]**	**Alive at 2 months** (*n* = 151)		**Lost to follow-up** (*n* = 9)		***p*-value**
IL-6 (pg/mL)	18.7	[5.2 – 40.4]	17.8	[6.2 – 34.8]	0.79
sTNFR-1 (ng/mL)	1.85	[1.23 – 2.73]	2.41	[1.28 – 3.51]	0.57
sTNFR-2 (ng/mL)	5.23	[3.47 – 7.83]	8.20	[3.80 – 14.60]	0.41
hsCRP (µg/mL)	31.6	[9.8 – 89.1]	32.2	[13.1 – 98.0]	0.76
Fibrinogen (mg/mL)	7.32	[4.25 – 11.69]	6.80	[4.77 – 8.98]	0.98
D-dimer (ng/mL)	905	[473 – 1,780]	975	[476 – 1134]	0.63
sCD27 (U/mL)	43.3	[21.5 – 73.9]	53.0	[44.2 – 61.0]	0.30
IP-10 (ng/mL)	0.75	[0.39 – 1.46]	0.57	[0.19 – 0.76]	0.26
sCD14 (µg/mL)	2.77	[2.11 – 3.80]	3.37	[2.69 – 5.05]	0.13
sCD163 (ng/mL)	858	[567 – 1,399]	945	[576 - 1,544]	0.76
Hyaluronan (ng/mL)	44.1	[25.6 – 87.9]	44.7	[26.2 – 290.1]	0.70
IFABP (ng/mL)	0.86	[0.46 – 1.49]	0.59	[0.30 – 3.63]	0.75

Differences were tested by Mann-Whitney *U* Test.
