# Supplementary material for: HIV infection is associated with elevated biomarkers of immune activation in Ugandan adults with pneumonia
Source: PLoS One. 2019 May 15;14(5):e0216680. doi: 10.1371/journal.pone.0216680 (PMC6519791; doi:10.1371/journal.pone.0216680)
Supplement: S4 Table — (PDF) [file pone.0216680.s004.pdf]

S4 Table. Risk ratios, with 95% confidence intervals, comparing the risk of mortality for the highest tertile compared to lower tertiles for each biomarker, stratified by HIV

Biomarker	Risk Ratio HIV-uninfected	Risk Ratio HIV-infected	*p*-value*	Crude Combined Risk Ratio	Mantel-Haenszel Combined Risk Ratio
Interleukin 6	3.50 (0.53, 23.14)	3.83 (0.82, 17.88)	0.94	4.04 (1.27, 12.8)	3.72 (1.11, 12.4)
Soluble TNF Receptor 1	4.79 (0.74, 31.16)	3.95 (0.87, 17.90)	0.87	4.71 (1.52, 14.6)	4.17 (1.25, 13.89)
Soluble TNF Receptor 2	4.79 (0.74, 31.16)	2.37 (0.63, 8.84)	0.54	3.44 (1.18, 10.0)	2.85 (0.97, 8.37)
High sensitivity C-reactive protein	3.50 (0.53, 22.93)	8.69 (1.13, 66.66)	0.50	6.13 (1.74, 21.6)	6.10 (1.49, 24.99)
Fibrinogen	4.67 (0.45, 48.71)	0.47 (0.11, 2.11)	0.11	0.98 (0.31, 3.08)	0.90 (0.30, 2.74)
D-dimer	0.95 (0.10, 8.66)	1.13 (0.33, 3.90)	0.89	1.23 (0.42, 3.57)	1.08 (0.37, 3.19)
Soluble CD27	0	0.85 (0.21, 3.50)	0.69	0.86 (0.23, 3.16)	0.64 (0.16, 2.61)
IFN-γ-Inducible Protein 10	4.06 (0.62, 26.68)	3.76 (0.83, 17.05)	0.95	4.34 (1.40, 13.5)	3.85 (1.16, 12.78)
Soluble CD14	2.67 (0.31, 23.00)	7.09 (0.93, 54.2)	0.49	4.71 (1.52, 14.6)	5.38 (1.14, 25.31)
Soluble CD163	2.38 (0.35, 15.90)	3.59 (0.97, 13.28)	0.73	3.29 (1.13, 9.57)	3.16 (1.08, 9.23)
Hyaluronan	1.92 (0.22, 16.93)	3.59 (0.79, 16.25)	0.64	3.35 (1.15, 9.75)	3.07 (0.88, 10.68)
Intestinal Fatty Acid Binding Protein	0.71 (0.07, 7.43)	1.53 (0.43, 5.46)	0.57	1.01 (0.32, 3.18)	1.23 (0.41, 3.75)

* *p*-value for the chi-squared test for homogeneity
